# Racial/Ethnic and Geographic Variations In Long-Term Survival Among Medicare Beneficiaries After Acute Ischemic Stroke

**DOI:** 10.5888/pcd18.200242

**Published:** 2021-02-18

**Authors:** Xin Tong, Linda Schieb, Mary G. George, Cathleen Gillespie, Robert K. Merritt, Quanhe Yang

**Affiliations:** 1 Division for Heart Disease and Stroke Prevention, National Center for Chronic Disease Prevention and Health Promotion, Centers for Disease Control and Prevention, Atlanta, Georgia

## Abstract

**Introduction:**

Little information is available about racial/ethnic and geographic variations in long-term survival among older patients (≥65) after acute ischemic stroke (AIS).

**Methods:**

We examined data on 1,019,267 Medicare fee-for-service (FFS) beneficiaries aged 66 or older, hospitalized with a primary diagnosis of AIS from 2008 through 2012. Survival was defined as the time from the date of AIS to date of death, or an end of follow-up date of December 31, 2017. We used Cox proportional hazard models to estimate 5-year survival after AIS, adjusted for age, sex, race and Hispanic ethnicity, poverty level, Charlson Comorbidity Index, and state.

**Results:**

Among 1,019,267 Medicare FFS beneficiaries hospitalized with AIS from 2008 through 2012, we documented 701,718 deaths (68.8%) during a median of 4 years of follow-up with 4.08 million person-years. The overall adjusted 5-year survival was 44%. Non-Hispanic Black men had the lowest 5-year survival, and 5-year survival varied significantly by state, from the highest at 49.1% (North Dakota) to the lowest at 40.5% (Hawaii). The ranges between the highest and lowest 5-year survival rates across states also varied significantly by racial/ethnic groups, with percentage point differences of 9.6 among non-Hispanic White, 11.3 among non-Hispanic Black, 17.7 among Hispanic, and 28.5 among other racial/ethnic beneficiaries.

**Conclusion:**

We identified significant racial/ethnic and geographic variations in 5-year survival rates after AIS among 2008–2012 Medicare FFS beneficiaries. Further study is needed to understand the reasons for these variations and develop prevention strategies to improve survival and racial disparities in survival after AIS.

**Summary**

**What is already known about this subject?**

Many studies showed a racial/ethnic disparity in stroke risk factors, hospitalizations, incidence, and mortality among older patients with stroke.

**What is added by this report?**

We assessed the long-term survival of older patients after hospitalization with acute ischemic stroke and identified the significant racial/ethnic and geographic variations.

**What are the implications for public health practice?**

Prevention strategies need to be developed to reduce the disparities in stroke treatment and access to health care, especially among minority racial/ethnic groups.

## Introduction

Stroke is the fifth leading cause of death in the United States with approximately 795,000 new or recurrent acute strokes occurring every year. The annual direct medical cost for stroke was estimated at $30.8 billion from 2016 through 2017 (1). Although stroke risks and mortality have declined considerably, racial/ethnic and geographic disparities remain significant (1). Recent studies suggest that the decline in stroke mortality stalled in recent years and that demographic and geographic variations remained substantial (2,3). However, limited studies examined the long-term survival after stroke and racial/ethnic and geographic variations in stroke survival among older adults (defined as ≥65 y) in the United States.

The aim of our study was to assess long-term (5-year) survival among patients aged 66 or older after acute ischemic stroke (AIS) and to examine racial/ethnic differences and geographic variations in stroke survival. Our findings may provide information to improve survival and reduce survival disparities after stroke among older adults in the United States.

## Methods

### Data sources and study sample

We used Medicare’s enrollment databases to generate our study cohort among Medicare fee-for-service (FFS) beneficiaries and Medicare Provider Analysis and Review (MEDPAR) data to assess overall survival among beneficiaries hospitalized with AIS from 2008 through 2012. To select the final analytical cohort we 1) identified all Medicare FFS beneficiaries aged 65 or older with 12 months continuous enrolment in Medicare parts A and B during 2007–2012; 2) identified all hospitalizations with AIS as the primary diagnosis among FFS beneficiaries from 2007 through 2012, including multiple admissions; and 3) used a 12-month or longer lookback period to identify the first AIS hospitalization. The length of lookback time varied by the years of Medicare enrollment; for example, 12 months for beneficiaries aged 66 (Medicare eligible at age 65 years), 24 months for those aged 67, and so on. Because of the 12-month or longer lookback period, our final cohort included FFS beneficiaries aged 66 or older with AIS hospitalizations from 2008 through 2012 (2007 served as lookback time). We used MEDPAR files to identify AIS, our outcome of interest. The MEDPAR files contained records for inpatient hospital stays and skilled nursing facility stays for all Medicare beneficiaries, and we used the primary diagnosis codes (International Classification of Diseases, 9th revision [ICD-9-CM] [4] codes 433.01, 433.11, 433.21, 433.31, 433.81, 433.91, 434.01, 434.11, and 434.91) to identify beneficiaries with AIS. We excluded all institutional long-term stay hospitalizations. We identified 1,019,267 FFS beneficiaries aged 66 or older in our study period who had AIS. Socioeconomic status (SES) in the community, defined by the percentage below the poverty level in the county of beneficiary residence in 2008, was linked to Medicare data from the Health Resources and Services Administration Area Health Resources Files (https://data.hrsa.gov/data/download).

### Statistical methods

We examined differences in the distribution of demographic features by χ^2^ test for categorical variables, and *t* test for continuous variables. The 5-year survival was defined as the time from the date of AIS to the date of death, or the date of end of follow-up (December 31, 2017), whichever came first. We used the National Death Index linked to Medicare data available through the Centers for Medicare and Medicaid Services (CMS) to determine the date of death. We performed 5-year survival analyses and subgroup analyses by age groups (66–74, 75–84, and ≥85), sex, race and Hispanic ethnicity (non-Hispanic White, non-Hispanic Black, Hispanic, and other non-Hispanic races), and SES at the county level (quartile distribution; higher quartiles indicate higher level of poverty). We identified Charlson Comorbidity Index (CCI) conditions (5) by using secondary diagnosis codes. We examined the variations in AIS survival across the states for all beneficiaries and by race and Hispanic ethnicity. Univariate and multivariate survival analyses of 5-year survival after AIS were carried out using the Kaplan–Meier life table, and Cox proportional hazards regression analyses adjusting for age, sex, race and Hispanic ethnicity, SES, state (Model 1); and for CCI (0, 1, 2, 3, and ≥4) (Model 2). For subgroup analyses, we defined insufficient data if the total events (deaths) per analytic group were fewer than 15 during follow-up. We used SAS, version 9.4 (SAS Institute) for analyses and considered a 2-sided *P* value of <.05 significant. Medicare data are available from CMS, US Department of Health and Human Services, for any qualified investigator.

## Results

From 2008 through 2012, AIS was the primary reason for hospitalization of 1,019,267 Medicare FFS beneficiaries (Table 1). Their median age at AIS admission was 79.9 (interquartile range [IQR], 73.5–85.8), 31% were aged 66–74, 41% were 75–84, and 28% were 85 or older. Forty-four percent of those FFS beneficiaries were men and 84% were non-Hispanic White. A quarter of AIS beneficiaries had no comorbidity as defined by CCI, and 14% had 4 or more comorbidities. Compared with other racial/ethnic groups, non-Hispanic Black AIS beneficiaries had a higher percentage of those who were aged 66 to 74 (41%), women (61%), had household incomes 75% below the poverty level (41%), or had 4 or more CCI comorbidity conditions (21%).

**Table 1:** Characteristics of Medicare Fee-for-Service Beneficiaries Aged ≥66 Admitted to Hospital With Acute Ischemic Stroke, Medicare Cohort 2008–2017^a^

Variable	Overall	Non-Hispanic White	Non-Hispanic Black	Hispanic	Other^b^
**Total**	1,019,267 (100.0)	856,648 (84.0)	94,001 (9.2)	43,278 (4.2)	25,340 (2.5)
**Age, y, median (IQR)**	79.9 (73.5–85.8)	80.2 (73.9–86.1)	77.2 (71.4–83.9)	78.2 (72.3–84.1)	78.6 (72.6–84.6)
**Age, y**					
66–74	312,294 (30.6)	249,424 (29.1)	38,306 (40.8)	15,703 (36.3)	8,861 (35.0)
75–84	419,128 (41.1)	354,990 (41.4)	35,467 (37.7)	18,162 (42.0)	10,509 (41.5)
≥85	287,845 (28.2)	252,234 (29.4)	20,228 (21.5)	9,413 (21.8)	5,970 (23.6)
**Sex**					
Male	451,296 (44.3)	383,081 (44.7)	36,396 (38.7)	19,991 (46.2)	11,828 (46.7)
Female	567,971 (55.7)	473,567 (55.3)	57,605 (61.3)	23,287 (53.8)	13,512 (53.3)
**Socioeconomic status^c^**, %					
≤25	260,798 (25.6)	231,553 (27.0)	14,520 (15.4)	6,302 (14.6)	8,423 (33.2)
26–50	254,642 (25.0)	226,558 (26.4)	13,942 (14.8)	8,287 (19.1)	5,855 (23.1)
51–75	261,930 (25.7)	213,565 (24.9)	27,430 (29.2)	14,341 (33.1)	6,594 (26.0)
>75	241,897 (23.7)	184,972 (21.6)	38,109 (40.5)	14,348 (33.2)	4,468 (17.6)
**Charlson Comorbidity Index**					
0	254,247 (24.9)	224,783 (26.2)	15,891 (16.9)	8,055 (18.6)	5,518 (21.8)
1	246,124 (24.1)	209,321 (24.4)	20,220 (21.5)	10,631 (24.6)	5,952 (23.5)
2	225,019 (22.1)	189,608 (22.1)	20,541 (21.9)	9,201 (21.3)	5,669 (22.4)
3	151,140 (14.8)	121,737 (14.2)	17,620 (18.7)	7,633 (17.6)	4,150 (16.4)
≥4	142,737 (14.0)	111,199 (13.0)	19,729 (21.0)	7,758 (17.9)	4,051 (16.0)
**Death**	701,718 (68.8)	591,493 (69.0)	66,172 (70.4)	28,239 (65.3)	15,814 (62.4)

Abbreviation: IQR, interquartile range.

a Values are number (percentage) unless otherwise indicated.

b Other non-Hispanic races.

c Socioeconomic status was defined by percentage below poverty level in the county of beneficiary residence in 2008; higher quartiles indicated higher level of poverty.

Overall, 701,718 (68.8%) beneficiaries with AIS died after hospitalization during a median of 4.0 years follow-up with a total of 4.08 million person-years. Crude overall 5-year survival was 43.7%, and adjusted survival was 44.1% (Model 1) and 44.0% (Model 2) (Table 2). The adjusted 5-year survival rate decreased significantly with increasing age and was similar for men and women (44%). We saw noticeable differences in survival by race and Hispanic ethnicity and county-level SES. Non-Hispanic Black beneficiaries had the lowest crude 5-year survival (41.4%) but had a comparable adjusted 5-year survival compared with non-Hispanic White beneficiaries (43.6% vs 43.8%, Model 2); Hispanic and other races/ethnicities remained stable compared with the crude estimates. By looking at sex-specific estimates by race and Hispanic ethnicity, non-Hispanic Black men (40.8%) and non-Hispanic White women (43.4%) had the lowest adjusted survival (Model 2) compared with the people of other races/ethnicities and Hispanic ethnicity. The 5-year survival rate decreased as county levels of poverty increased.

**Table 2:** Crude and Adjusted 5-Year Survival After Acute Ischemic Stroke Among Medicare Fee-for-Service Beneficiaries Aged ≥66, Medicare Cohort 2008–2017^a^

Characteristic	Crude^b^	Adjusted Model 1^c^	Adjusted Model 2^d^
**Total** e	43.7 (43.6–43.8)	44.1 (44.0–44.2)	44.0 (43.9–44.1)
**Age at acute ischemic stroke, y**			
66–74	64.2 (64.0–64.4)	64.7 (64.6–64.9)	63.7 (63.6–63.9)
75–84	45.6 (45.4–45.7)	45.8 (45.7–46.0)	45.4 (45.2–45.5)
≥85	18.9 (18.7–19.0)	19.3 (19.2–19.5)	20.7 (20.6–20.8)
**Sex**			
Men	46.9 (46.8–47.1)	44.0 (43.9–44.1)	44.2 (44.1–44.4)
Women	41.2 (41.1–41.3)	44.3 (44.2–44.4)	43.9 (43.7–44.0)
**Race/ethnicity**			
Non-Hispanic White	43.7 (43.6–43.8)	44.5 (44.4–44.6)	43.8 (43.7–43.9)
Non-Hispanic Black	41.4 (41.1–41.7)	40.1 (39.9–40.4)	43.6 (43.3–43.8)
Hispanic	46.3 (45.8–46.7)	44.5 (44.2–44.9)	46.6 (46.2–47.0)
Other non-Hispanic races	48.9 (48.3–49.5)	47.4 (46.9–48.0)	48.7 (48.3–49.2)
**Sex by race/ethnicity**			
**Men**			
Non-Hispanic White	47.2 (47.0–47.3)	44.6 (44.5–44.7)	44.3 (44.2–44.4)
Non-Hispanic Black	42.1 (41.6–42.6)	36.9 (36.5–37.3)	40.8 (40.4–41.2)
Hispanic	48.7 (48.0–49.4)	44.3 (43.7–44.9)	46.6 (46.1–47.1)
Other non-Hispanic races	51.6 (50.7–52.5)	47.4 (46.7–48.2)	48.9 (48.2–49.7)
**Women**			
Non-Hispanic White	40.9 (40.8–41.1)	44.4 (44.3–44.5)	43.4 (43.3–43.5)
Non-Hispanic Black	40.9 (40.5–41.3)	42.1 (41.7–42.4)	45.1 (44.8–45.4)
Hispanic	44.2 (43.5–44.8)	44.7 (44.2–45.3)	46.6 (46.1–47.1)
Other non-Hispanic races	46.5 (45.7–47.4)	47.5 (46.8–48.2)	48.6 (47.9–49.3)
**Socioeconomic status^f^, %**			
≤25	44.3 (44.1–44.5)	45.2 (45.0–45.4)	44.8 (44.7–45.0)
26–50	44.2 (44.0–44.4)	44.2 (44.0–44.3)	44.0 (43.9–44.2)
51–75	43.6 (43.4–43.8)	44.0 (43.9–44.2)	44.0 (43.9–44.2)
>75	42.8 (42.6–43.0)	43.1 (43.0–43.3)	43.2 (43.0–43.3)

a Values are percentage (95% CI).

b Crude survival was estimated by using Kaplan–Meier life table.

c Model 1 adjusted survivals were estimated using Cox proportional hazards analyses adjusting for age, sex, race and Hispanic ethnicity, socioeconomic status, and state.

d Model 2 includes Charlson Comorbidity Index (0, 1, 2, 3 and ≥4) in addition to the covariates in adjusted Model 1.

e The median follow-up time for all Medicare fee-for-service beneficiaries with acute ischemic stroke was 4.0 years with a total of 4.08 million person-years.

f Socioeconomic status was defined by percentage below poverty level in the county of beneficiary residence in 2008; higher quartiles indicated higher level of poverty.

**Figure 1:**
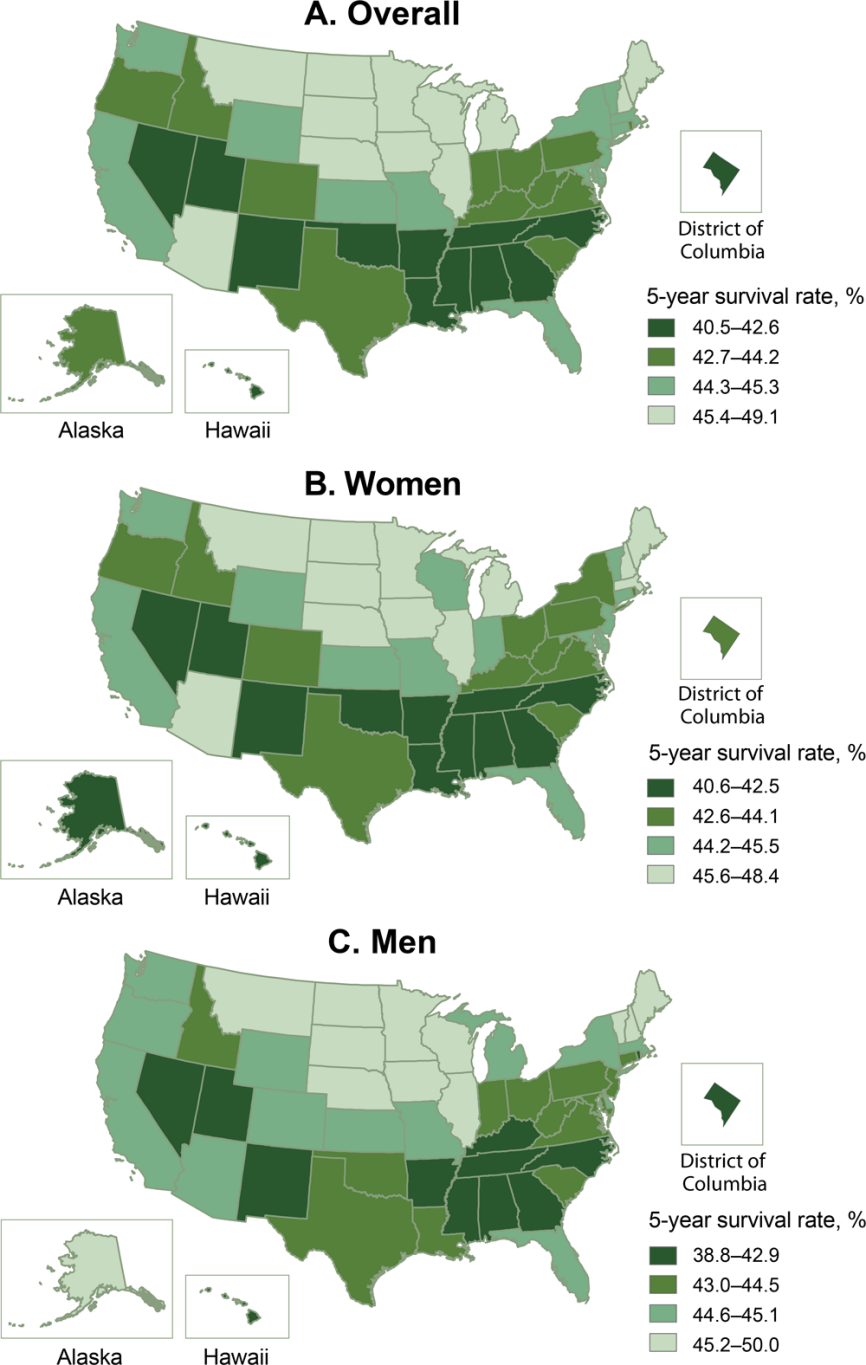
Adjusted 5-year survival after acute ischemic stroke among Medicare fee-for-service beneficiaries, Medicare cohort 2008–2017. Map A shows the adjusted 5-year survival after acute ischemic stroke among all Medicare fee-for-service beneficiaries. Map B shows the adjusted 5-year survival among women, and Map C shows the adjusted 5-year survival among men.

**Figure 2:**
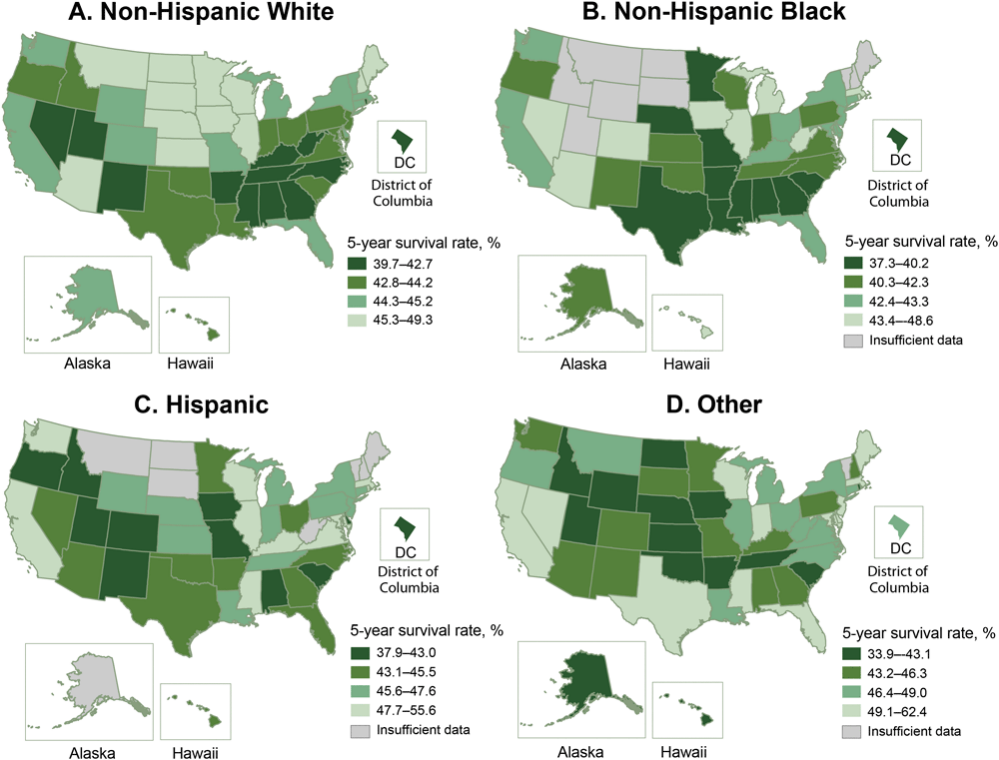
Adjusted 5-year survival after acute ischemic stroke by race and Hispanic ethnicity among Medicare fee-for-service beneficiaries, Medicare cohort 2008–2017. Map A shows the adjusted 5-year survival after acute ischemic stroke among non-Hispanic White Medicare beneficiaries. Map B shows the adjusted 5-year survival among non-Hispanic Black beneficiaries. Map C shows the adjusted 5-year survival among Hispanic Medicare beneficiaries. Map D shows the adjusted 5-year survival among other (other non-Hispanic races) Medicare beneficiaries. Abbreviation: —, insufficient data.

The adjusted 5-year survival rates following AIS varied significantly across the states. Hawaii had the lowest 5-year survival rate (40.5%), Alabama had the second lowest (40.8%), and North Dakota (49.1%) and South Dakota (48.6%) had the highest (Figure 1) (Table 3). Several stroke belt (6) and southern states (Alabama, Arkansas, Georgia, Kentucky, Louisiana, Mississippi, North Carolina, and Tennessee) were among the states with the 15 lowest survival rates (range 40.8%– 42.7%). The lowest survival rate observed among non-Hispanic Black beneficiaries was in some states in the Midwest and the Southeast, and the highest survival rates were among states in the West and Northeast (Figure 2). However, for non-Hispanic White beneficiaries, the highest survival rates were in the Midwestern states, and the lowest survival rates were mainly in the Southeast. The survival pattern for Hispanic beneficiaries and those of other races/ethnicities was different from that of non-Hispanic White and non-Hispanic Black beneficiaries, with the lowest survival rates scattered outside of the Southeast. We saw substantial differences in 5-year survival rates across the states among each race and Hispanic ethnicity. Among non-Hispanic White groups, survival rates ranged from the highest, 49.3%, in North Dakota to the lowest, 39.7%, in the District of Columbia, a 9.6 percentage point difference. For non-Hispanic Black groups, it ranged from 48.6% in Arizona to 37.3% in Minnesota, an 11.3 percentage point difference. For Hispanic groups, the rate was 55.6% in Mississippi and 37.9% in Delaware, with a 17.7 percentage point difference. Other races/ethnicities had a difference of 28.5 percentage points in survival rates across the states, with the highest rate in Delaware, 62.4%, and the lowest rate in Idaho, 33.9%.

**Table:** 

State	Adjusted 5-Year Survival, %		
	Overall	Men	Women
Alabama	40.8	40.5	41.1
Alaska	43.4	45.2	41.9
Arizona	45.5	45.1	45.9
Arkansas	40.8	41.1	40.6
California	44.8	45.1	44.7
Colorado	44.2	44.7	43.9
Connecticut	44.6	44.5	44.7
Delaware	44.7	44.8	44.7
District of Columbia	42.3	39.6	43.3
Florida	44.6	44.8	44.5
Georgia	42.4	42.7	42.3
Hawaii	40.5	38.8	41.8
Idaho	43.1	43.2	43.2
Illinois	45.5	45.4	45.6
Indiana	44.2	44.1	44.4
Iowa	47.3	47.2	47.5
Kansas	45.3	45.1	45.5
Kentucky	42.7	41.8	43.5
Louisiana	42.6	43.3	42.1
Maine	45.8	45.8	46.0
Maryland	44.5	44.3	44.8
Massachusetts	45.2	44.9	45.5
Michigan	45.3	45.0	45.5
Minnesota	47.4	47.5	47.4
Mississippi	42.1	41.9	42.2
Missouri	44.4	44.8	44.2
Montana	46.6	47.2	46.1
Nebraska	46.1	45.4	46.7
Nevada	42.3	42.2	42.5
New Hampshire	46.6	47.5	46.0
New Jersey	44.2	44.2	44.4
New Mexico	41.3	41.8	40.9
New York	44.5	45.1	44.1
North Carolina	42.4	42.5	42.4
North Dakota	49.1	50.0	48.3
Ohio	44.0	44.2	43.9
Oklahoma	42.4	43.1	42.0
Oregon	44.1	44.6	43.8
Pennsylvania	44.0	43.9	44.1
Rhode Island	42.6	42.9	42.5
South Carolina	43.2	43.6	43.1
South Dakota	48.6	49.0	48.4
Tennessee	42.0	41.6	42.3
Texas	43.2	43.4	43.1
Utah	42.3	42.3	42.3
Vermont	45.0	45.5	44.7
Virginia	43.3	43.5	43.3
Washington	44.6	44.7	44.6
West Virginia	42.8	43.2	42.6
Wisconsin	45.8	46.5	45.5
Wyoming	45.0	44.9	45.1

**Table 3:** Demographic Information and Adjusted 5-Year Survival After Acute Ischemic Stroke Among Medicare Fee-for-Service Beneficiaries by State, Medicare Cohort 2008–2017

State	Overall 5-year Survival^a^, % (95% CI)	Non-Hispanic White		Non-Hispanic Black		Hispanic		Other^b^	
		% of Cohort^c^	5-year Survival^a^, % (95% CI)	% of cohort^c^	5-year Survival^a^, % (95% CI)	% of Cohort^c^	5-year Survival^a^, % (95% CI)	% of Cohort^c^	5-year Survival^a^, % (95% CI)
Alabama	40.8 (40.3–41.3)	83.1	40.8 (40.3–41.4)	16.1	38.2 (36.9–39.5)	0.3	39.7 (31.7–49.9)	0.5	43.4 (36.5–51.5)
Alaska	43.4 (41.3–45.7)	77.9	44.3 (41.9–46.8)	2.6	40.9 (29.1–57.4)	2.0	—	17.5	42.8 (37.8–48.4)
Arizona	45.5 (44.9–46.1)	89.1	45.6 (45.0–46.3)	1.9	48.6 (44.2–53.4)	5.9	45.0 (42.5–47.7)	3.1	46.3 (42.8–49.9)
Arkansas	40.8 (40.2–41.4)	90.1	40.8 (40.2–41.4)	8.6	37.7 (35.6–39.9)	0.5	44.0 (35.7–54.3)	0.8	40.9 (34.2–48.8)
California	44.8 (44.5–45.1)	69.2	44.3 (44.0–44.7)	6.2	42.8 (41.6–43.9)	14.1	47.9 (47.1–48.8)	10.5	50.7 (49.7–51.6)
Colorado	44.2 (43.4–45.0)	88.0	44.6 (43.8–45.5)	2.7	43.8 (38.9–49.3)	7.6	40.6 (37.8–43.6)	1.8	44.3 (38.6–50.8)
Connecticut	44.6 (44.0–45.2)	90.6	44.7 (44.0–45.3)	5.1	42.6 (39.7–45.6)	2.8	45.6 (41.7–49.8)	1.4	47.1 (41.8–53.1)
Delaware	44.7 (43.6–45.8)	83.6	44.3 (43.2–45.6)	13.6	43.3 (40.2–46.6)	1.1	37.9 (29.1–49.4)	1.7	62.4 (54.2–71.8)
District of Columbia	42.3 (40.7–44.0)	23.2	39.7 (36.4–43.2)	72.9	40.2 (38.1–42.3)	1.9	42.9 (31.9–57.8)	2.0	47.2 (36.5–61.1)
Florida	44.6 (44.3–44.9)	84.2	44.8 (44.5–45.1)	6.9	42.4 (41.3–43.6)	7.7	44.3 (43.2–45.4)	1.2	51.1 (48.5–53.8)
Georgia	42.4 (42.0–42.8)	80.0	42.4 (41.9–42.9)	18.4	40.0 (39.0–41.1)	0.8	44.3 (39.3–49.9)	0.8	45.9 (41.0–51.3)
Hawaii	40.5 (39.0–42.0)	26.8	43.8 (41.0–46.7)	1.0	45.4 (32.7–63.1)	5.8	45.0 (39.1–51.9)	66.4	42.3 (40.3–44.4)
Idaho	43.1 (41.9–44.4)	96.1	43.5 (42.2–44.7)	0.2	—	1.9	42.9 (34.4–53.4)	1.8	33.9 (26.3–43.6)
Illinois	45.5 (45.1–45.8)	83.8	45.5 (45.2–45.9)	11.3	43.4 (42.4–44.6)	3.3	48.7 (46.8–50.7)	1.6	47.1 (44.3–50.1)
Indiana	44.2 (43.8–44.7)	91.2	44.2 (43.7–44.6)	6.8	42.3 (40.6–44.2)	1.3	46.2 (42.4–50.4)	0.6	50.1 (44.3–56.5)
Iowa	47.3 (46.6–48.0)	97.4	47.3 (46.7–48.0)	1.3	45.2 (39.4–51.9)	0.6	42.4 (34.4–52.2)	0.7	42.9 (35.3–52.2)
Kansas	45.3 (44.6–45.9)	93.6	45.5 (44.8–46.2)	3.6	41.0 (37.5–44.9)	1.6	46.0 (40.9–51.7)	1.2	43.1 (37.0–50.1)
Kentucky	42.7 (42.2–43.3)	94.7	42.6 (42.1–43.1)	4.7	42.4 (39.9–45.0)	0.2	52.2 (41.5–65.7)	0.4	44.1 (35.5–54.7)
Louisiana	42.6 (42.0–43.1)	74.3	43.0 (42.3–43.6)	23.4	38.8 (37.6–40.1)	1.5	46.6 (42.1–51.5)	0.8	48.6 (42.2–56.1)
Maine	45.8 (44.9–46.8)	98.8	45.9 (45.0–46.9)	0.2	—	0.2	—	0.8	49.5 (38.2–64.0)
Maryland	44.5 (44.0–45.0)	75.1	44.2 (43.7–44.8)	21.3	42.7 (41.6–43.9)	1.3	52.0 (47.7–56.8)	2.4	51.0 (47.6–54.7)
Massachusetts	45.2 (44.7–45.7)	91.7	45.0 (44.5–45.5)	3.5	47.8 (45.0–50.7)	2.7	49.3 (46.3–52.6)	2.2	52.1 (48.7–55.8)
Michigan	45.3 (44.9–45.6)	85.4	45.1 (44.7–45.5)	12.1	44.1 (43.0–45.2)	1.2	45.9 (42.7–49.4)	1.3	47.9 (44.7–51.4)
Minnesota	47.4 (46.7–48.1)	96.9	47.6 (46.9–48.4)	1.2	37.3 (31.4–44.3)	0.5	45.5 (35.7–57.8)	1.4	44.5 (38.8–50.9)
Mississippi	42.1 (41.5–42.7)	75.7	42.0 (41.3–42.7)	23.3	39.1 (37.8–40.4)	0.3	55.6 (45.3–68.3)	0.6	51.1 (43.1–60.5)
Missouri	44.4 (43.9–44.9)	92.1	44.6 (44.1–45.1)	6.8	40.0 (38.2–42.0)	0.6	38.2 (32.4–45.0)	0.6	44.8 (38.6–52.0)
Montana	46.6 (45.3–47.9)	94.6	46.8 (45.5–48.1)	0.3	—	0.8	—	4.3	46.5 (40.5–53.4)
Nebraska	46.1 (45.2–47.1)	95.6	46.3 (45.4–47.3)	2.0	38.3 (32.2–45.7)	1.4	46.7 (39.1–55.9)	1.0	40.9 (32.0–52.4)
Nevada	42.3 (41.4–43.3)	82.6	41.9 (40.8–42.9)	6.3	43.3 (39.4–47.6)	6.1	44.5 (40.5–48.9)	5.0	50.9 (46.5–55.8)
New Hampshire	46.6 (45.5–47.6)	98.2	46.7 (45.6–47.8)	0.3	—	0.7	—	0.8	43.4 (32.7–57.6)
New Jersey	44.2 (43.8–44.6)	82.0	44.1 (43.7–44.5)	10.5	42.5 (41.2–43.8)	5.2	47.6 (45.8–49.4)	2.3	50.2 (47.5–53.1)
New Mexico	41.3 (40.2–42.3)	69.0	41.4 (40.2–42.7)	1.6	40.3 (32.3–50.3)	24.4	42.9 (40.7–45.2)	4.9	45.1 (40.2–50.5)
New York	44.5 (44.2–44.8)	80.5	44.3 (44.0–44.7)	10.1	42.9 (41.9–44.0)	5.9	47.4 (46.0–48.8)	3.5	49.0 (47.3–50.9)
North Carolina	42.4 (42.0–42.8)	81.2	42.3 (41.9–42.8)	16.8	40.2 (39.3–41.2)	0.6	43.5 (38.6–49.0)	1.4	46.5 (43.1–50.1)
North Dakota	49.1 (47.6–50.6)	97.7	49.3 (47.8–50.8)	0.0	—	0.2	—	2.0	41.4 (32.3–53.0)
Ohio	44.0 (43.6–44.3)	89.8	43.9 (43.6–44.3)	8.6	42.8 (41.5–44.2)	0.9	45.1 (41.3–49.2)	0.7	47.1 (42.8–51.9)
Oklahoma	42.4 (41.8–43.0)	86.9	42.7 (42.1–43.3)	4.4	41.8 (39.0–44.7)	1.2	44.8 (39.6–50.6)	7.5	42.1 (40.0–44.3)
Oregon	44.1 (43.3–44.9)	94.8	44.2 (43.4–45.0)	0.9	41.1 (33.7–50.1)	1.8	41.3 (35.8–47.8)	2.5	47.2 (42.1–53.0)
Pennsylvania	44.0 (43.6–44.3)	92.1	44.1 (43.7–44.4)	5.8	40.4 (39.0–42.0)	1.1	46.6 (43.3–50.2)	1.0	46.3 (42.7–50.2)
Rhode Island	42.6 (41.3–44.0)	92.3	42.4 (41.1–43.9)	2.4	46.3 (37.4–57.5)	3.6	48.3 (41.0–56.9)	1.8	46.1 (35.6–59.8)
South Carolina	43.2 (42.7–43.8)	80.3	43.6 (43.0–44.2)	18.7	39.5 (38.2–40.8)	0.5	43.0 (35.7–51.8)	0.5	42.8 (35.8–51.2)
South Dakota	48.6 (47.3–50.0)	95.4	48.8 (47.5–50.2)	0.3	—	0.3	—	4.0	45.3 (39.1–52.4)
Tennessee	42.0 (41.5–42.4)	89.2	41.9 (41.4–42.4)	9.9	40.2 (38.7–41.8)	0.4	45.6 (38.1–54.6)	0.5	42.9 (36.5–50.4)
Texas	43.2 (42.9–43.4)	74.8	43.3 (42.9–43.6)	8.8	39.7 (38.8–40.7)	14.0	45.1 (44.2–46.0)	1.7	49.8 (47.7–52.1)
Utah	42.3 (41.2–43.5)	94.5	42.5 (41.3–43.7)	0.4	—	3.1	42.0 (35.5–49.7)	2.0	41.8 (34.3–51.0)
Vermont	45.0 (43.4–46.7)	98.6	45.0 (43.4–46.6)	0.2	—	0.4	—	0.8	—
Virginia	43.3 (42.9–43.7)	81.0	43.3 (42.9–43.8)	16.0	40.6 (39.5–41.8)	0.9	48.5 (44.0–53.4)	2.1	46.6 (43.5–49.9)
Washington	44.6 (44.0–45.1)	90.6	44.7 (44.1–45.2)	2.0	43.2 (39.3–47.4)	2.1	50.6 (46.8–54.7)	5.3	44.6 (42.2–47.1)
West Virginia	42.8 (42.0–43.6)	97.1	42.7 (41.9–43.5)	2.2	43.7 (38.4–49.6)	0.2	—	0.4	46.7 (35.4–61.6)
Wisconsin	45.8 (45.3–46.4)	94.5	45.9 (45.4–46.5)	3.1	41.5 (38.3–45.0)	1.1	48.9 (43.7–54.7)	1.4	49.5 (44.8–54.8)
Wyoming	45.0 (43.2–46.9)	93.8	45.2 (43.3–47.2)	0.4	—	3.4	47.2 (37.8–59.1)	2.3	34.3 (24.0–49.1)

Abbreviation: —, insufficient data.

a Adjusted survivals were estimated by using Cox proportional hazards analyses adjusting for age, sex, race and Hispanic ethnicity, socioeconomic status, and Charlson Comorbidity Index.

b Other non-Hispanic races.

c Percentage of total acute ischemic stroke Medicare fee-for-service beneficiaries with acute ischemic stroke, from 2008 through 2012.

**Table:** 

State	Adjusted 5-year Survival by Race/Ethnicity			
	Non-Hispanic White	Non-Hispanic Black	Hispanic	Other
Alabama	40.8	38.2	39.7	43.4
Alaska	44.3	40.9	—	42.8
Arizona	45.6	48.6	45.0	46.3
Arkansas	40.8	37.7	44.0	40.9
California	44.3	42.8	47.9	50.7
Colorado	44.6	43.8	40.6	44.3
Connecticut	44.7	42.6	45.6	47.1
Delaware	44.3	43.3	37.9	62.4
District of Columbia	39.7	40.2	42.9	47.2
Florida	44.8	42.4	44.3	51.1
Georgia	42.4	40.0	44.3	45.9
Hawaii	43.8	45.4	45.0	42.3
Idaho	43.5	—	42.9	33.9
Illinois	45.5	43.4	48.7	47.1
Indiana	44.2	42.3	46.2	50.1
Iowa	47.3	45.2	42.4	42.9
Kansas	45.5	41.0	46.0	43.1
Kentucky	42.6	42.4	52.2	44.1
Louisiana	43.0	38.8	46.6	48.6
Maine	45.9	—	—	49.5
Maryland	44.2	42.7	52.0	51.0
Massachusetts	45.0	47.8	49.3	52.1
Michigan	45.1	44.1	45.9	47.9
Minnesota	47.6	37.3	45.5	44.5
Mississippi	42.0	39.1	55.6	51.1
Missouri	44.6	40.0	38.2	44.8
Montana	46.8	—	—	46.5
Nebraska	46.3	38.3	46.7	40.9
Nevada	41.9	43.3	44.5	50.9
New Hampshire	46.7	—	—	43.4
New Jersey	44.1	42.5	47.6	50.2
New Mexico	41.4	40.3	42.9	45.1
New York	44.3	42.9	47.4	49.0
North Carolina	42.3	40.2	43.5	46.5
North Dakota	49.3	—	—	41.4
Ohio	43.9	42.8	45.1	47.1
Oklahoma	42.7	41.8	44.8	42.1
Oregon	44.2	41.1	41.3	47.2
Pennsylvania	44.1	40.4	46.6	46.3
Rhode Island	42.4	46.3	48.3	46.1
South Carolina	43.6	39.5	43.0	42.8
South Dakota	48.8	—	—	45.3
Tennessee	41.9	40.2	45.6	42.9
Texas	43.3	39.7	45.1	49.8
Utah	42.5	—	42.0	41.8
Vermont	45.0	—	—	—
Virginia	43.3	40.6	48.5	46.6
Washington	44.7	43.2	50.6	44.6
West Virginia	42.7	43.7	—	46.7
Wisconsin	45.9	41.5	48.9	49.5
Wyoming	45.2	—	47.2	34.3

## Discussion

Our study’s findings suggested that about 2 in 5 Medicare FFS beneficiaries aged 66 or older survived at least 5 years after hospitalization for AIS. Men and women had similar 5-year survival. We found significant racial/ethnic and geographic variations in 5-year survival after AIS. Non-Hispanic Black men had the lowest adjusted 5-year survival. Non-Hispanic White beneficiaries overall had the least variation in adjusted 5-year survival across states; other races/ethnicities had the greatest variation.

Many studies reported racial disparities in stroke risk factors and in stroke hospitalizations, incidence, and mortality (7–9), but few focused on long-term survival after stroke. An early study using Medicare data suggested that non-Hispanic Black people aged 65 or older, especially men, had significantly lower survival after stroke than non-Hispanic White people, consistent with our findings (10). Yao et al recently reported that Black Medicare beneficiaries were at higher risk for ischemic stroke than White beneficiaries and more likely to have diabetes or obesity (7). The Northern Manhattan Stroke study suggested that Black and Caribbean Hispanic people had more stroke risk factors than White people in their community-based multiethnic population study (8). The Reasons for Geographic and Racial Differences in Stroke (REGARDS) study reported that Black people had a greater age- and sex-adjusted mean 10-year predicted stroke risk than White people, which contributed to disparities in stroke mortality (9). Reports from the REGARDS study suggested that although management of acute stroke appeared to be more equivalent between Black and White participants, the racial disparity in stroke mortality was largely driven by differences in stroke incidence (11). Stroke mortality mainly depends on the incidence of stroke associated with the stroke risk profiles in a population (11,12), and stroke survival depends on prestroke morbidity and frailty, comorbid conditions, severity of stroke, access to stroke treatment, and quality of care (13,14). Therefore, a population with a higher stroke risk profile, incidence, and mortality could have a better survival rate after stroke than those from a population with lower stroke incidence and mortality. Our findings showed that the crude difference in survival between non-Hispanic White and non-Hispanic Black populations, especially among women, became insignificant after adjusting for demographics, SES, and CCI, suggesting the importance of prestroke comorbidities (Model 1 vs Model 2) in explaining racial differences in stroke survival. Further studies are needed to examine the relative contribution of stroke risk factors, prestroke morbidity and frailty, treatments, and care to racial disparities in stroke survival.

Our study found that Medicare FFS beneficiaries in the southeastern United States region had the lowest 5-year survival following AIS. The findings of recent studies showed significant geographic variations in stroke death rates at the county level, and in the long-established stroke belt in the Southeast (15,16). In addition, a study based on 2000–2002 Medicare FFS beneficiaries discharged with an incident ischemic stroke reported that the highest recurrent stroke rates occurred in the southern regions (17).

Our study suggested that the differences in 5-year survival after AIS across the states appeared to be wider for Hispanic people and other races compared with non-Hispanic White and non-Hispanic Black people. The difference between the highest and the lowest survival rates across the states ranged from 9.6 to 28.5 percentage points by race and Hispanic ethnicity. Reasons for these significant differences are not clear. Among Hispanic beneficiaries, the top 5 highest 5-year survival rates were in Massachusetts (49.3%), Washington (50.6%), Maryland (52.0%), Kentucky (52.2%), and Mississippi (55.6%), whereas the 5 lowest survival rates were in Oregon (41.3%), Colorado (40.6%), Alabama (39.7%), Missouri (38.2%), and Delaware (37.9%). With the rapid growth of the Hispanic population in the United States (18,19), there may be a gap in assessing stroke risk factors, access to health care, and promoting stroke prevention programs across the states among Hispanic residents. Samet et al reported a notably high proportion of Hispanic adults in Texas with obesity and diabetes (20). The study, which was conducted between 2008 and 2011 and included 15,079 Hispanic participants, reported the pervasive burden of cardiovascular disease risk factors among Hispanic participants and identified the risk factors (hypertension, diabetes, and smoking) associated with stroke (21). Other studies reported significant disparities in stroke care among racial/ethnic minority groups compared with White participants (22).

A recent CMS report noted that disparities in clinical care among Hispanic and non-Hispanic White populations varied greatly by geography, especially in rural areas (23). Although these geographic disparities were not related to stroke care, they may contribute to the wider variations in access to stroke care and survival across the states among Hispanic residents. A few studies also explored the differences in stroke outcomes between non-Hispanic White people and Hispanic, Asian American, and Chinese people (24–27). A study of participants with AIS over age 65 in the American Heart Association’s Get With The Guidelines–Stroke program found that non-Hispanic Black and Hispanic patients had higher adjusted 1-year all-cause rehospitalization than non-Hispanic White patients (24). A study conducted in Hawaii comparing potentially preventable 30-day readmissions after stroke found that Chinese patients may be at higher risk than non-Hispanic White patients (25). One Medicare study found that beneficiaries in hospitals with stroke certification had lower stroke mortality, regardless of the size of the hospital, than hospitals without certification (26). Another Get With The Guidelines–Stroke study with linked Medicare data showed that academic hospitals as compared with nonacademic hospitals and those in the Northeast or West compared with South or Midwest had more favorable stroke outcomes (27). The higher stroke risk profile, pre-stroke comorbidities, stroke severity, differences in access to health care after stroke, and stroke prevention programs may contribute to the wider variations in 5-year survival after AIS among minority groups across the states. In addition, minority beneficiaries may be underrepresented among Medicare FFS beneficiaries, which may contribute to the wider variation in 5-year stroke survival and limit the generalizability of our findings to minority beneficiaries (28,29).

Our study had limitations. First, because of the lack of measures of stroke severity, we were unable to examine its impact on overall survival. Second, AIS hospitalizations and deaths were based on administrative records and limited to Medicare FFS beneficiaries aged 66 or older. The first AIS hospitalizations identified in the MEDPAR database might not in fact be the first if the beneficiaries had a stroke before they enrolled in Medicare. Third, the AIS diagnosis was based on ICD-9-CM codes from claims data and was not clinically verified, which could lead to possible misclassification. Fourth, the wider variations in 5-year stroke survival rates observed among Hispanic people and people of other races/ethnicities may be due to the limited sample size for these groups. Lastly, the findings based on FFS beneficiaries in our study may not be generalizable to Medicare patients covered under a health maintenance organization (HMO) plan because of the possible differences in beneficiary characteristics between the 2 types of coverage plans.

Our findings demonstrated significant racial/ethnic and geographic differences in long-term survival after AIS. The variations across states in different racial/ethnic groups call for further study addressing disparities in treatment and access to health care, especially among minority groups. Stroke outcomes could be improved through public health and clinical strategies, such as awareness of risk factors, early diagnosis, and aggressive management of risk factors. Further research may explain the reasons for the significant geographic variations in survival after AIS and help develop prevention strategies to reduce these gaps across the states.
